# Supplementary material for: Bayesian generalized method of moments applied to pseudo-observations in survival analysis
Source: Lifetime Data Anal. 2025 Sep 22;31(4):970–93. doi: 10.1007/s10985-025-09670-1 (PMC12586244; doi:10.1007/s10985-025-09670-1)
Supplement: Supplementary file 1 — (pdf 764 KB) [file 10985_2025_9670_MOESM1_ESM.pdf]

Supporting Information for: Bayesian generalized method of moments applied to pseudo-observations in survival analysis

Table S1a: Performances of the GMM models compared to Cox, GEE, and piecewise exponential (PEM) models with different sample sizes. The true log hazard ratio is fixed at -0.1 (HR=0.9) and the censoring rate at 20%.

n	Methods	Bias	ASE	ASD	RMSE	Coverage
**50**	**Frequentist**					
	Cox	-0.0141	0.325	0.328	0.329	94.8
	GEE	-0.0058	0.353	0.382	0.382	92.2
	GMM	-0.0058	0.365	0.382	0.382	93.4
	**Bayesian**					
	PEM	-0.0288	0.331	0.361	0.362	92.6
	GMM	-0.0927	0.350	0.377	0.388	91.1
**100**	**Frequentist**					
	Cox	0.0084	0.227	0.241	0.242	93.3
	GEE	0.0134	0.252	0.269	0.269	93.5
	GMM	0.0134	0.256	0.269	0.269	93.9
	**Bayesian**					
	PEM	-0.0002	0.232	0.261	0.261	91.2
	GMM	-0.0380	0.251	0.269	0.271	92.2
**200**	**Frequentist**					
	Cox	0.0004	0.159	0.159	0.159	94.0
	GEE	0.0013	0.179	0.189	0.189	93.3
	GMM	0.0013	0.180	0.189	0.189	93.7
	**Bayesian**					
	PEM	-0.0073	0.163	0.171	0.171	92.7
	GMM	-0.0064	0.187	0.196	0.196	92.8
**500**	**Frequentist**					
	Cox	0.0029	0.100	0.100	0.100	94.1
	GEE	0.0041	0.114	0.112	0.112	94.9
	GMM	0.0041	0.114	0.112	0.112	95.0
	**Bayesian**					
	PEM	-0.0006	0.101	0.103	0.103	94.0
	GMM	0.0012	0.116	0.113	0.113	94.9
**1000**	**Frequentist**					
	Cox	0.0028	0.071	0.072	0.072	95.0
	GEE	0.0014	0.080	0.080	0.080	95.5
	GMM	0.0014	0.080	0.080	0.080	95.5
	**Bayesian**					
	PEM	0.0011	0.071	0.073	0.073	94.4
	GMM	0.0000	0.081	0.081	0.081	95.3

ASE: Average Standard Error, ASD: Average Standard Deviation
RMSE: Root Mean Square Error

Table S1b: Performances of the GMM models compared to Cox, GEE, and piecewise exponential (PEM) models with different sample sizes. The true log hazard ratio is fixed at -0.5 (HR=0.6) and the censoring rate at 20%.

n	Methods	Bias	ASE	ASD	RMSE	Coverage
**50**	**Frequentist**					
	Cox	-0.0240	0.330	0.338	0.339	95.5
	GEE	-0.0200	0.359	0.395	0.396	92.4
	GMM	-0.0200	0.372	0.395	0.396	93.8
	**Bayesian**					
	PEM	-0.0802	0.335	0.367	0.376	92.0
	GMM	-0.0763	0.357	0.379	0.387	91.2
**100**	**Frequentist**					
	Cox	0.0026	0.230	0.250	0.250	93.7
	GEE	0.0076	0.256	0.276	0.276	94.2
	GMM	0.0076	0.260	0.276	0.276	94.4
	**Bayesian**					
	PEM	-0.0327	0.234	0.266	0.268	91.6
	GMM	-0.0295	0.256	0.273	0.275	92.5
**200**	**Frequentist**					
	Cox	-0.0017	0.161	0.162	0.162	94.4
	GEE	0.0002	0.182	0.190	0.190	92.9
	GMM	0.0002	0.183	0.190	0.190	93.1
	**Bayesian**					
	PEM	-0.0329	0.164	0.173	0.176	93.0
	GMM	-0.0239	0.192	0.198	0.199	93.0
**500**	**Frequentist**					
	Cox	0.0034	0.102	0.101	0.101	94.8
	GEE	0.0020	0.115	0.112	0.112	95.0
	GMM	0.0020	0.115	0.112	0.112	95.1
	**Bayesian**					
	PEM	-0.0107	0.102	0.104	0.104	94.1
	GMM	-0.0071	0.118	0.114	0.114	95.6
**1000**	**Frequentist**					
	Cox	0.0034	0.072	0.072	0.072	95.2
	GEE	0.0014	0.081	0.082	0.082	94.9
	GMM	0.0014	0.082	0.082	0.082	94.9
	**Bayesian**					
	PEM	-0.0039	0.072	0.074	0.074	94.9
	GMM	-0.0030	0.082	0.083	0.083	95.0

ASE: Average Standard Error, ASD: Average Standard Deviation
RMSE: Root Mean Square Error

Table S2a: Performances of the GMM models compared to Cox, GEE, and piecewise exponential (PEM) models with different censoring rates. The true log hazard ratio is fixed at -0.1 (HR=0.9) and the sample size at 500.

CR	Methods	Bias	ASE	ASD	RMSE	Coverage
**5%**	**Frequentist**					
	Cox	0.0023	0.092	0.093	0.093	94.3
	GEE	0.0029	0.106	0.104	0.105	95.5
	GMM	0.0029	0.107	0.104	0.105	95.5
	**Bayesian**					
	PEM	-0.0012	0.093	0.097	0.097	93.9
	GMM	0.0000	0.108	0.107	0.107	95.7
**10%**	**Frequentist**					
	Cox	0.0015	0.095	0.094	0.094	94.8
	GEE	0.0020	0.108	0.105	0.105	95.4
	GMM	0.0020	0.109	0.105	0.105	95.4
	**Bayesian**					
	PEM	-0.0022	0.096	0.098	0.098	93.8
	GMM	-0.0076	0.108	0.105	0.106	95.2
**20%**	**Frequentist**					
	Cox	0.0029	0.100	0.100	0.100	94.1
	GEE	0.0041	0.114	0.112	0.112	94.9
	GMM	0.0041	0.114	0.112	0.112	95.0
	**Bayesian**					
	PEM	-0.0006	0.101	0.103	0.103	94.0
	GMM	0.0012	0.116	0.113	0.113	94.9
**30%**	**Frequentist**					
	Cox	0.0050	0.107	0.106	0.107	95.4
	GEE	0.0052	0.120	0.117	0.117	95.5
	GMM	0.0052	0.121	0.117	0.117	95.7
	**Bayesian**					
	PEM	0.0014	0.108	0.110	0.110	94.7
	GMM	0.0023	0.123	0.119	0.119	95.4
**70%**	**Frequentist**					
	Cox	0.0035	0.164	0.163	0.163	95.1
	GEE	0.0018	0.183	0.184	0.184	94.9
	GMM	0.0018	0.184	0.184	0.184	94.9
	**Bayesian**					
	PEM	0.0008	0.165	0.169	0.169	94.2
	GMM	-0.0066	0.193	0.169	0.169	95.1

CR: Censoring Rate, ASE: Average Standard Error,
ASD: Average Standard Deviation, RMSE: Root Mean Square Error

Table S2b: Performances of the GMM models compared to Cox, GEE, and piecewise exponential (PEM) models with different censoring rates. The true log hazard ratio is fixed at -0.5 (HR=0.6) and the sample size at 500.

CR	Methods	Bias	ASE	ASD	RMSE	Coverage
**5%**	**Frequentist**					
	Cox	0.0015	0.094	0.096	0.096	94.2
	GEE	0.0041	0.108	0.108	0.108	95.2
	GMM	0.0041	0.108	0.108	0.108	95.4
	**Bayesian**					
	PEM	-0.0127	0.095	0.098	0.099	94.1
	GMM	-0.0062	0.111	0.110	0.111	94.9
**10%**	**Frequentist**					
	Cox	0.0015	0.096	0.097	0.097	94.4
	GEE	0.0035	0.110	0.108	0.108	95.2
	GMM	0.0035	0.110	0.108	0.108	95.2
	**Bayesian**					
	PEM	-0.0121	0.097	0.099	0.100	94.7
	GMM	-0.0064	0.113	0.111	0.111	95.0
**20%**	**Frequentist**					
	Cox	0.0034	0.102	0.101	0.101	94.8
	GEE	0.0020	0.115	0.112	0.112	95.0
	GMM	0.0020	0.115	0.112	0.112	95.1
	**Bayesian**					
	PEM	-0.0107	0.102	0.104	0.104	94.1
	GMM	-0.0071	0.118	0.114	0.114	95.6
**30%**	**Frequentist**					
	Cox	0.0047	0.108	0.109	0.109	94.9
	GEE	0.0016	0.122	0.121	0.121	94.9
	GMM	0.0016	0.123	0.121	0.121	94.9
	**Bayesian**					
	PEM	-0.0091	0.109	0.112	0.112	94.2
	GMM	-0.0075	0.125	0.121	0.123	94.9
**70%**	**Frequentist**					
	Cox	0.0020	0.167	0.165	0.165	94.5
	GEE	-0.0019	0.187	0.188	0.188	93.9
	GMM	-0.0019	0.188	0.188	0.188	94.1
	**Bayesian**					
	PEM	-0.0111	0.168	0.170	0.170	93.9
	GMM	-0.0185	0.198	0.190	0.191	94.6

CR: Censoring Rate, ASE: Average Standard Error,
ASD: Average Standard Deviation, RMSE: Root Mean Square Error

Table S3: Comparison of the performances of GEE and GMM models with different time points. The true log hazard ratio is fixed at -0.3 (HR=0.74), the censoring rate at 20%, and the sample size at 500.

Methods	K	Bias	ASE	ASD	RMSE	Coverage
**Frequentist**						
GEE	5	0.0032	0.114	0.112	0.112	95.4
	7	0.0024	0.112	0.110	0.110	95.3
	10	0.0029	0.111	0.109	0.109	95.3
GMM	5	0.0032	0.114	0.112	0.112	95.5
	7	0.0024	0.113	0.110	0.110	95.3
	10	0.0029	0.111	0.109	0.109	95.4
**Bayesian**						
GMM	5	-0.0028	0.116	0.113	0.113	95.4
	7	-0.0051	0.114	0.112	0.112	95.3
	10	-0.0063	0.113	0.112	0.112	95.0

ASE: Average Standard Error, ASD: Average Standard Deviation
RMSE: Root Mean Square Error

Table S4a: Comparison of the performances of GEE and GMM models with different correlation matrices: independence (IND), exchangeable (EXCH), and first-order auto-regressive (AR-1). The true log hazard ratio is fixed at -0.1 (HR=0.9), the censoring rate at 20%, and the sample size at 500.

Methods	WCM	Bias	ASE	ASD	RMSE	Coverage
**Frequentist**						
GEE	IND	0.0041	0.114	0.112	0.112	94.9
GEE	EXCH	0.0033	0.113	0.112	0.112	95.1
GEE	AR-1	0.0036	0.111	0.110	0.110	94.7
GMM	IND	0.0041	0.114	0.112	0.112	95.0
GMM	EXCH	0.0028	0.111	0.110	0.110	95.7
GMM	AR-1	0.0020	0.110	0.110	0.110	95.4
**Bayesian**						
GMM	IND	0.0012	0.116	0.113	0.113	94.9
GMM	EXCH	-0.0002	0.112	0.111	0.111	95.2
GMM	AR-1	-0.0009	0.113	0.112	0.112	94.6

WCM: Working Correlation Matrix, ASE: Average Standard Error,
ASD: Average Standard Deviation, RMSE: Root Mean Square Error

Table S4b: Comparison of the performances of GEE and GMM models with different correlation matrices: independence (IND), exchangeable (EXCH), and first-order auto-regressive (AR-1). The true log hazard ratio is fixed at -0.5 (HR=0.6), the censoring rate at 20%, and the sample size at 500.

Methods	WCM	Bias	ASE	ASD	RMSE	Coverage
**Frequentist**						
GEE	IND	0.0020	0.115	0.112	0.112	95.0
GEE	EXCH	0.0009	0.114	0.112	0.112	95.5
GEE	AR-1	0.0013	0.112	0.110	0.110	95.5
GMM	IND	0.0020	0.115	0.112	0.112	95.1
GMM	EXCH	-0.0021	0.112	0.110	0.111	95.2
GMM	AR-1	-0.0051	0.112	0.111	0.111	95.2
**Bayesian**						
GMM	IND	-0.0071	0.118	0.114	0.114	95.6
GMM	EXCH	-0.0105	0.114	0.112	0.113	95.2
GMM	AR-1	-0.0138	0.114	0.113	0.114	94.8

WCM: Working Correlation Matrix, ASE: Average Standard Error,
ASD: Average Standard Deviation, RMSE: Root Mean Square Error

Table S5a: Log hazard ratio and standard error of the treatment effect estimated by GEE and GMM with different correlation matrices: independence (IND), exchangeable (EXCH), and first-order auto-regressive (AR-1) in $R2_{loc}$ trial for event-free survival and overall survival

Methods	WCM	log(HR)	SE
Event-free survival			
**Frequentist**			
GEE	IND	-0.5073	0.226
GEE	EXCH	-0.5383	0.220
GEE	AR-1	-0.4903	0.219
GMM	IND	-0.5073	0.227
GMM	EXCH	-0.5457	0.223
GMM	AR-1	-0.4549	0.228
**Bayesian**			
GMM	IND	-0.5389	0.248
GMM	EXCH	-0.5143	0.241
GMM	AR-1	-0.5025	0.281
Overall survival			
**Frequentist**			
GEE	IND	-0.3995	0.240
GEE	EXCH	-0.4529	0.236
GEE	AR-1	-0.4246	0.235
GMM	IND	-0.3995	0.242
GMM	EXCH	-0.4736	0.241
GMM	AR-1	-0.4755	0.247
**Bayesian**			
GMM	IND	-0.4507	0.270
GMM	EXCH	-0.5132	0.264
GMM	AR-1	-0.5430	0.311

WCM: Working Correlation Matrix
SE: Standard Error

Table S5b: Log hazard ratio and standard errors of the treatment effect estimated by GEE and GMM with different correlation matrices: independence (IND), exchangeable (EXCH), and first-order auto-regressive (AR-1) in $R2_{\mathrm{pulm}}$ trial for event-free survival and overall survival

Methods	WCM	log(HR)	SE
Event-free survival			
**Frequentist**			
GEE	IND	-0.1641	0.190
GEE	EXCH	-0.2086	0.186
GEE	AR-1	-0.1756	0.184
GMM	IND	-0.1641	0.191
GMM	EXCH	-0.2100	0.188
GMM	AR-1	-0.1791	0.184
**Bayesian**			
GMM	IND	-0.1798	0.196
GMM	EXCH	-0.1956	0.197
GMM	AR-1	-0.1985	0.199
Overall survival			
**Frequentist**			
GEE	IND	0.0676	0.208
GEE	EXCH	0.0095	0.204
GEE	AR-1	0.0469	0.203
GMM	IND	0.0676	0.209
GMM	EXCH	0.0029	0.208
GMM	AR-1	0.0240	0.203
**Bayesian**			
GMM	IND	0.0424	0.221
GMM	EXCH	0.0000	0.227
GMM	AR-1	0.0030	0.226

WCM: Working Correlation Matrix

SE: Standard Error

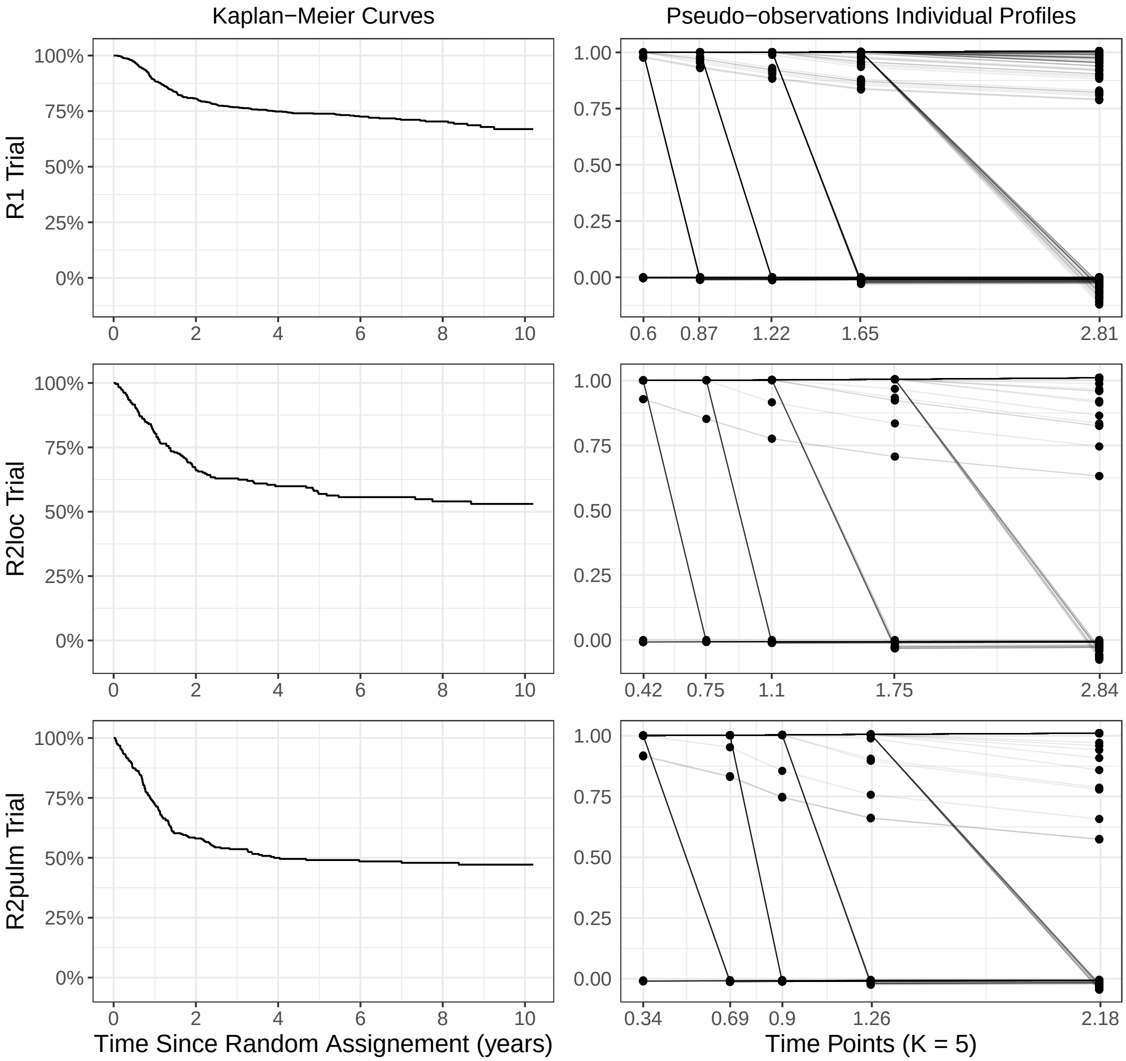

Figure S1: Kaplan-Meier curves of the event-free survival and corresponding pseudo-observations individual profiles for the EWING trials.

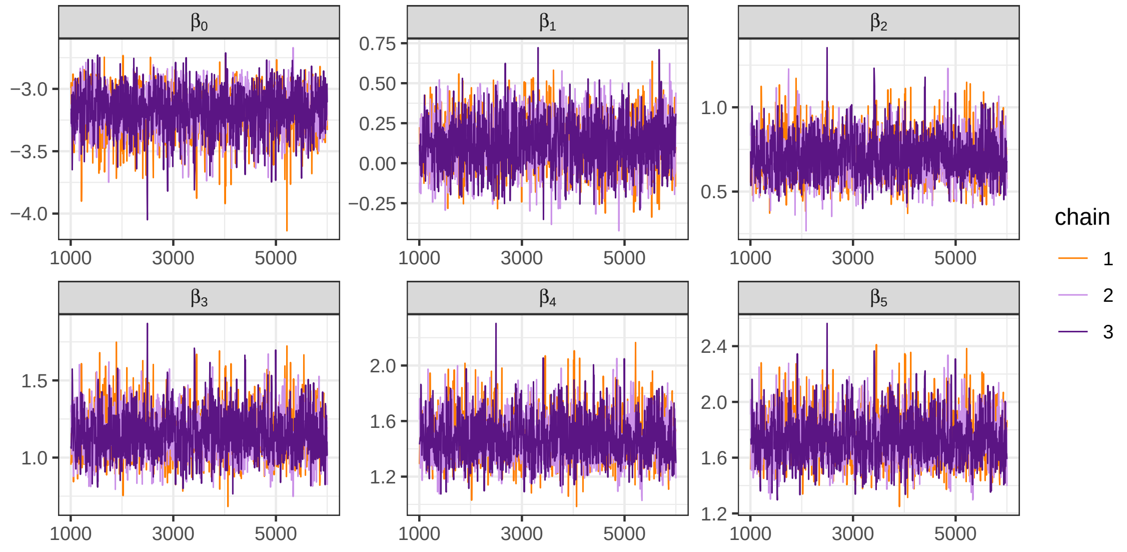

Figure S2a: Post warm-up MCMCs, using the Bayesian GMM to estimate HR based on the event-free survival in EWING (R1) trial. β_0 is the intercept, β_1 is the parameter of the treatment factor and $(\beta_2, ...\beta_5)$ are for the $K-1$ dummy time points variables.

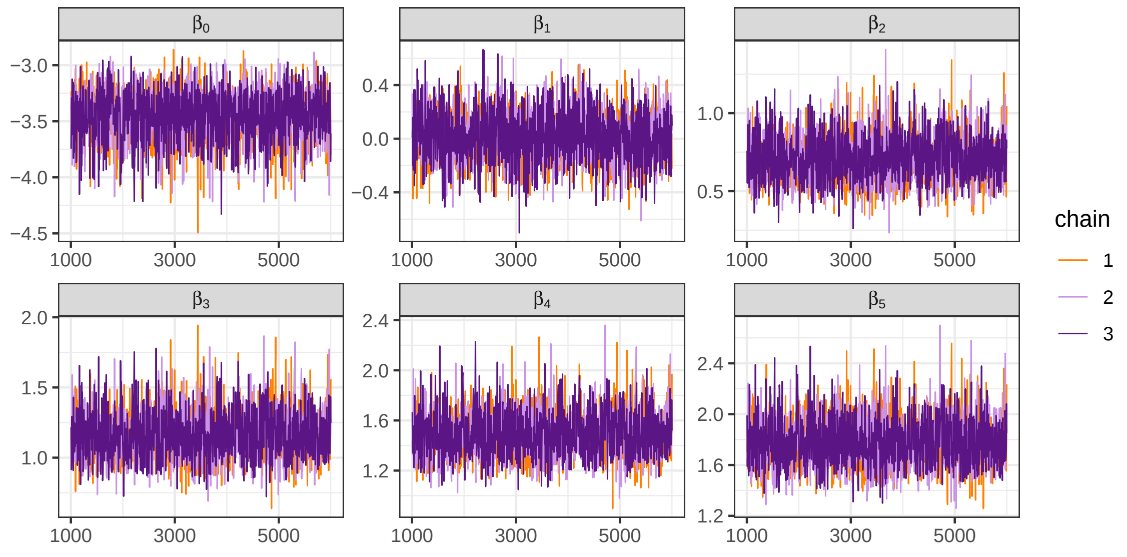

Figure S2b: Post warm-up MCMCs, using the Bayesian GMM to estimate HR based on the overall survival in EWING (R1) trial. β_0 is the intercept, β_1 is the parameter of the treatment factor and $(\beta_2, ...\beta_5)$ are for the $K-1$ dummy time points variables.

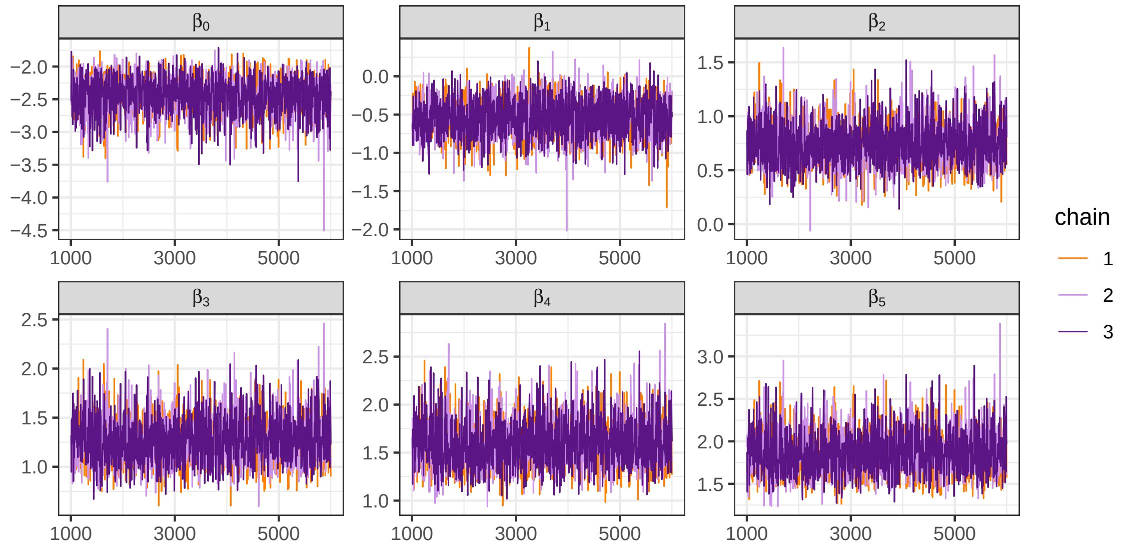

Figure S2c: Post warm-up MCMCs, using the Bayesian GMM to estimate HR based on the event-free survival in EWING ($R2_{loc}$) trial. β_0 is the intercept, β_1 is the parameter of the treatment factor and $(\beta_2, ...\beta_5)$ are for the $K-1$ dummy time points variables.

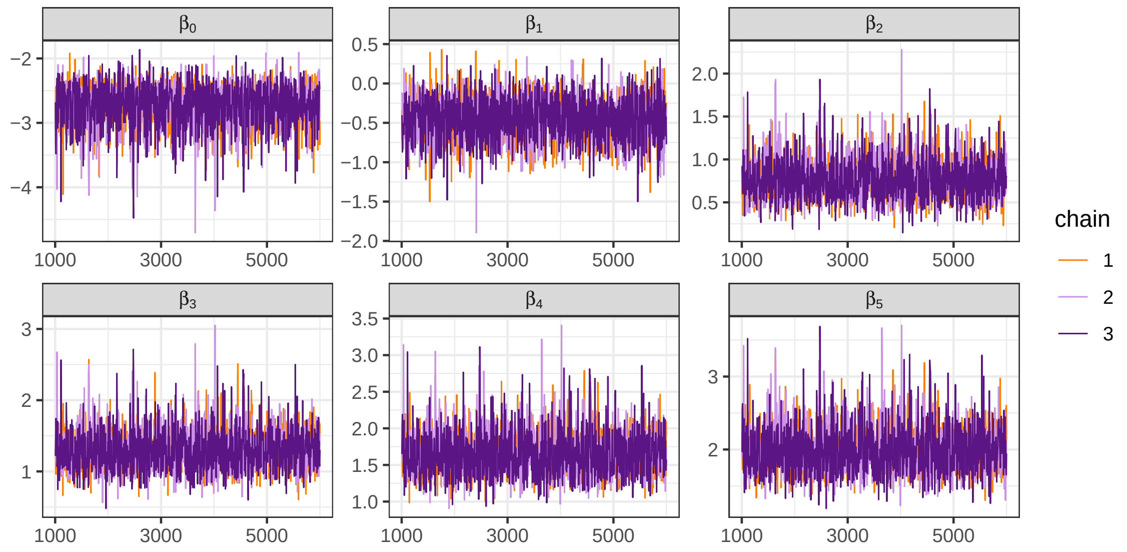

Figure S2d: Post warm-up MCMCs, using the Bayesian GMM to estimate HR based on the overall survival in EWING ($R2_{loc}$) trial. β_0 is the intercept, β_1 is the parameter of the treatment factor and $(\beta_2, ...\beta_5)$ are for the $K-1$ dummy time points variables.

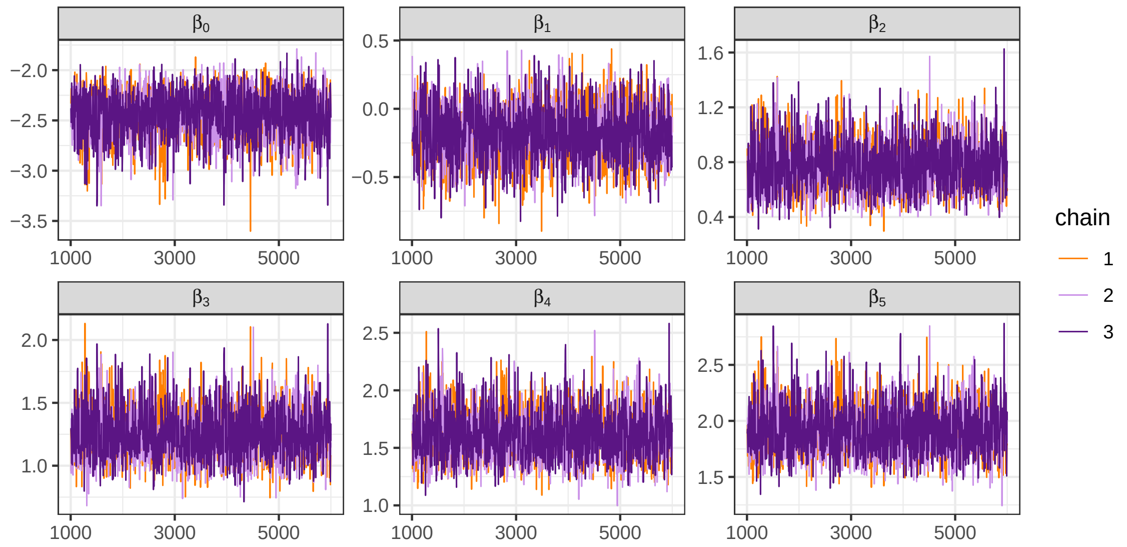

Figure S2e: Post warm-up MCMCs, using the Bayesian GMM to estimate HR based on the event-free survival in EWING ($R2_{pulm}$) trial. β_0 is the intercept, β_1 is the parameter of the treatment factor and $(\beta_2, ...\beta_5)$ are for the $K-1$ dummy time points variables.

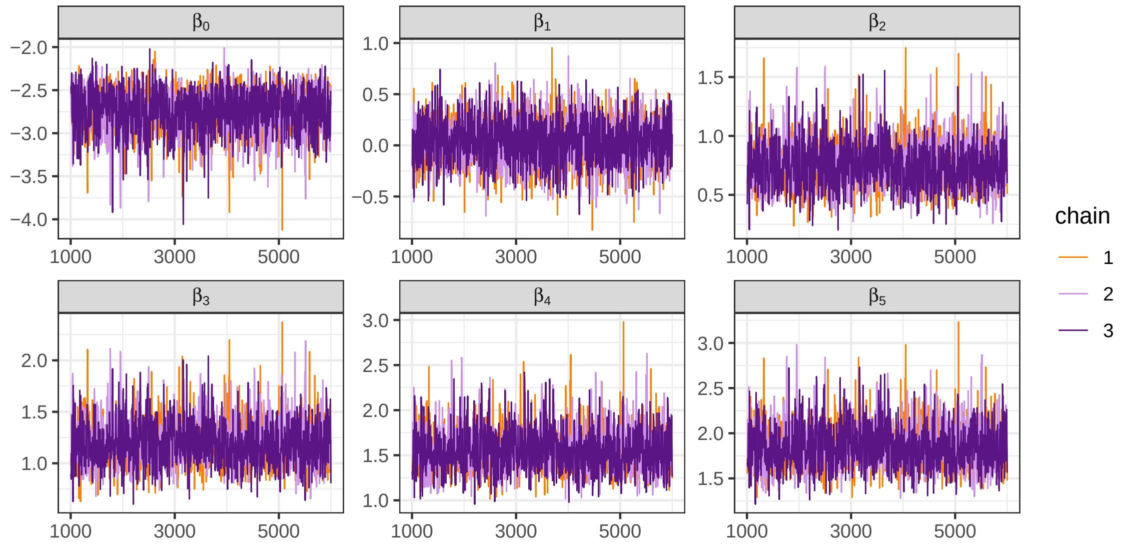

Figure S2f: Post warm-up MCMCs, using the Bayesian GMM to estimate HR based on the overall survival in EWING ($R2_{pulm}$) trial. β_0 is the intercept, β_1 is the parameter of the treatment factor and $(\beta_2, ...\beta_5)$ are for the $K-1$ dummy time points variables.

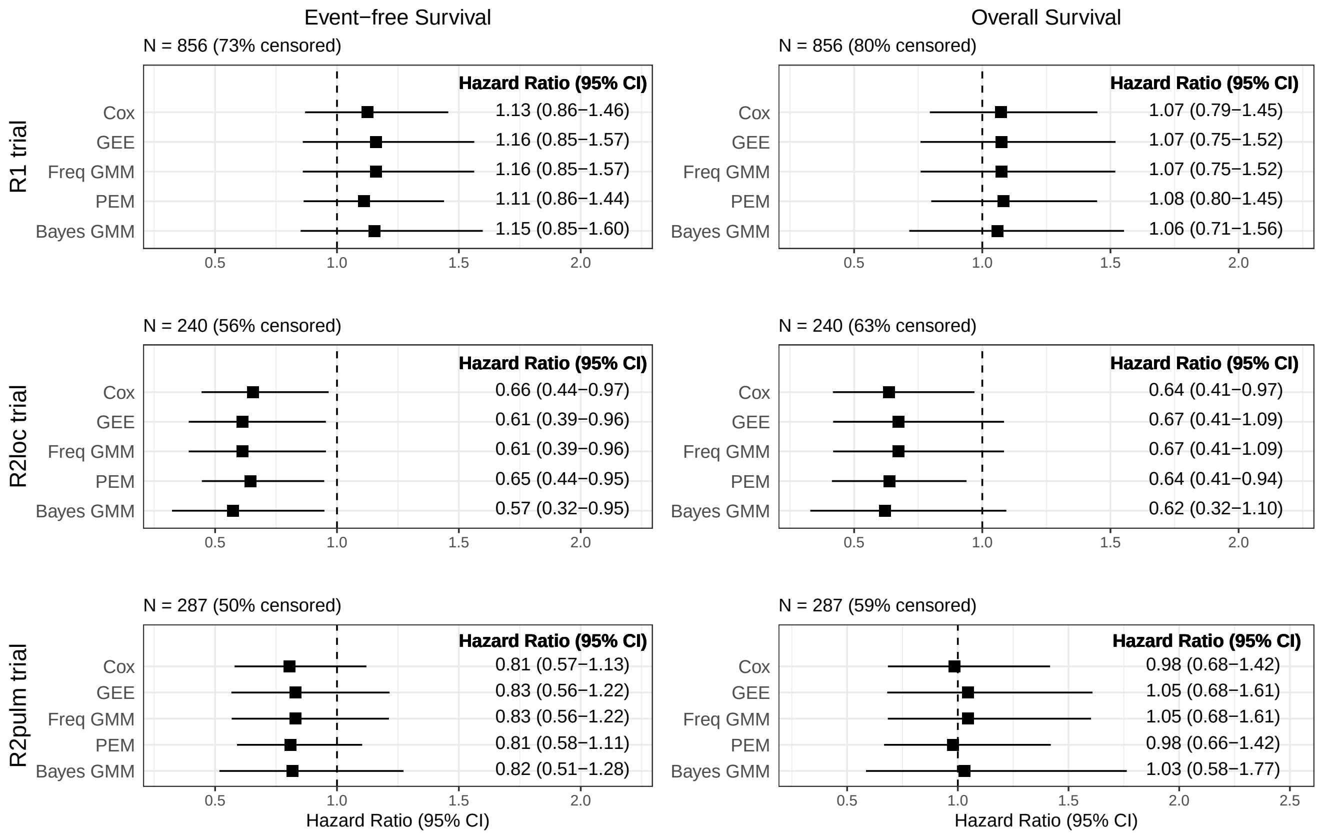

Figure S3: Hazard ratio estimates (and 95% confidence intervals) from the Cox proportional hazard, GEE, frequentist (Freq) GMM, piecewise exponential and Bayesian (Bayes) GMM models in the three EWING trials (R1, $R2_{\text{loc}}$, or $R2_{\text{pulm}}$) for event-free survival (left part) and overall survival (right part). These analyses were adjusted on the age variable: binary variable (< 25, ≥ 25) years in the R1 trial and categorical variable (< 12, $12-18$, $18-25$, > 25) years in the $R2_{\text{loc}}$, and $R2_{\text{pulm}}$ trials. The independent working correlation matrix is used for GEE and GMM approaches. The vertical dashed line represents the null effect.

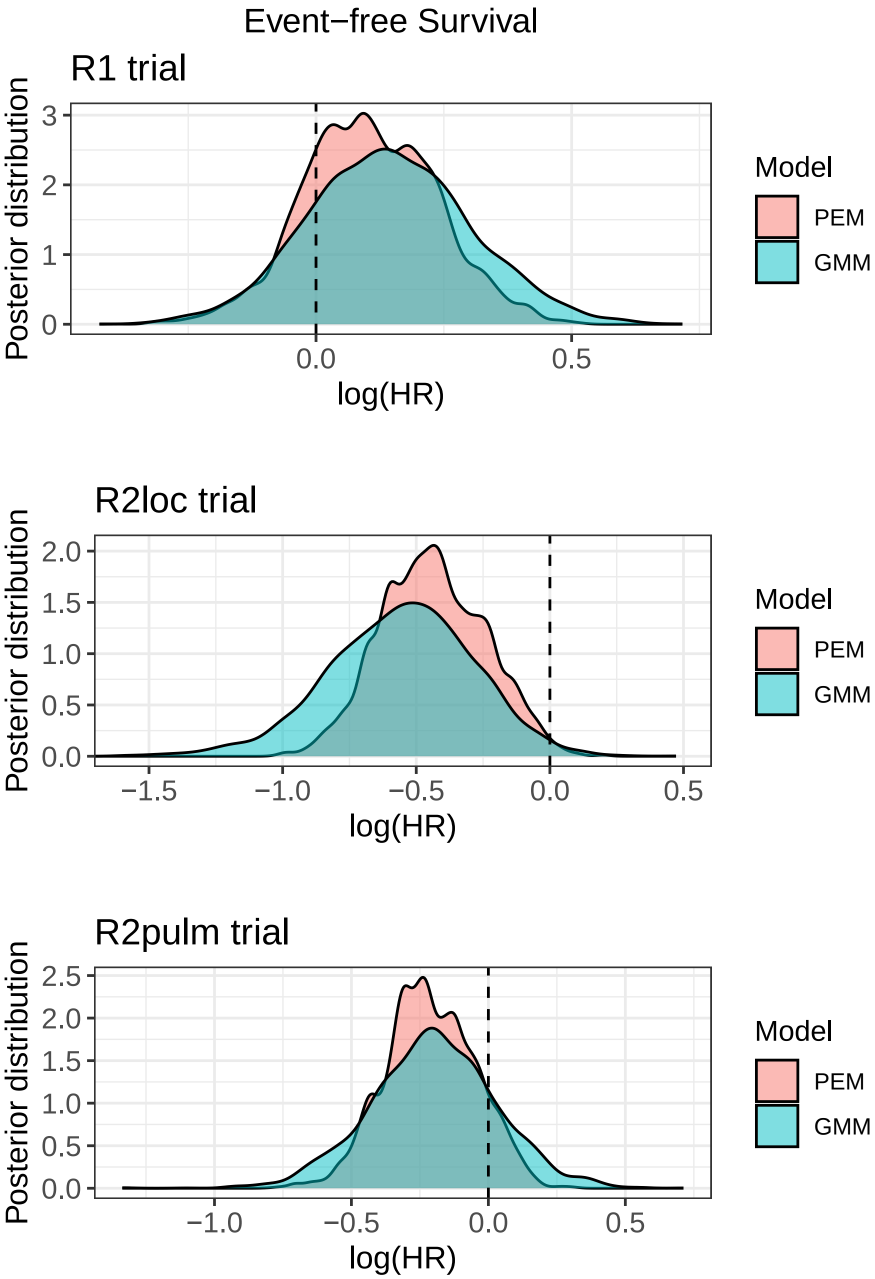

Figure S4: Posterior distribution of the log hazard ratios estimated with the piecewise exponential model (PEM) and the Bayesian generalized method of moments (GMM) in the three EWING trials (R1, $R2_{loc}$, or $R2_{pulm}$) for event-free survival. These analyses were adjusted on the age variable: binary variable ($< 25, \geq 25$) years in the R1 trial and categorical variable (< 12, $12 - 18$, $18 - 25$, > 25) years in the $R2_{loc}$, and $R2_{pulm}$ trials. The independent working correlation matrix is used for GEE and GMM approaches. The vertical dashed line represents the null effect.
